# 

**Published:** 1917-12

**Authors:** 


### ANNOUNCEMENTS

CLINIC CHICAGO DENTAL SOCIETY. The annual clinic of this society will be held at La Salle Hotel, in Chicago, January 26, 1918. It promises to be unusually good. Write Dr. T. A. Broadbent, for program.

MINNESOTA STATE DENTAL SOCIETY will hold its annual meeting February 7 to 9, at the University of Minnesota, in Minneapolis. All members of the National Dental Association will be cordially welcomed. Write Dr. Max E. Ernst, 1125 Lowry Buiding, St. Paul, Minnesota, for program.

A WARNING. The following letter sent to secretaries of state dental societies is self-explanatory and important:

"It has been brought to the notice of the government at Washington that men within the conscription age have made application to dentists to have teeth extracted to the extent of incapaciating them for military service. Orders have been issued to the state and local societies to inform the members that such cases are to be immediately reported to Dr. T. A. Broadbent, 25 E. Washington Street, secretary of Chicago Dental Society, giving name and address if possible of such persons.

"It is also demanded that men within the conscription age who have teeth extracted be prepared to produce the extracted teeth on request of the exemption board.

"Dentists who are guilty of extracting for this purpose will be prosecuted to the fullest extent by the Federal Government.

"Notify your members immediately upon receipt of this letter." Fraternally yours, T. A. BROADBENT, *Secretary,* Chicago Dental Society.

